# Cortical dynamics of disfluency in adults who stutter

**DOI:** 10.14814/phy2.13194

**Published:** 2017-05-08

**Authors:** Ranit Sengupta, Shalin Shah, Torrey M. J. Loucks, Kristin Pelczarski, J. Scott Yaruss, Katie Gore, Sazzad M. Nasir

**Affiliations:** ^1^ Department of Communication Sciences and DisordersNorthwestern UniversityEvanstonIllinois; ^2^ Department of Speech and Hearing ScienceUniversity of Illinois at Urbana‐ChampaignChampaignIllinois; ^3^ School of Family Studies and Human ServicesKansas State UniversityManhattanKansas; ^4^ Department of Communication Sciences and DisordersUniversity of PittsburghPittsburghPennsylvania; ^5^ Speech IRLChicagoIllinois

**Keywords:** Disfluent speech, neural oscillations, phase coherence, stuttering

## Abstract

Stuttering is a disorder of speech production whose origins have been traced to the central nervous system. One of the factors that may underlie stuttering is aberrant neural miscommunication within the speech motor network. It is thus argued that disfluency (any interruption in the forward flow of speech) in adults who stutter (AWS) could be associated with anomalous cortical dynamics. Aberrant brain activity has been demonstrated in AWS in the absence of overt disfluency, but recording neural activity during disfluency is more challenging. The paradigm adopted here took an important step that involved overt reading of long and complex speech tokens under continuous EEG recording. Anomalies in cortical dynamics preceding disfluency were assessed by subtracting out neural activity for fluent utterances from their disfluent counterparts. Differences in EEG spectral power involving alpha, beta, and gamma bands, as well as anomalies in phase‐coherence involving the gamma band, were observed prior to the production of the disfluent utterances. These findings provide novel evidence for compromised cortical dynamics that directly precede disfluency in AWS.

## Introduction

Stuttering is a communication disorder that negatively impacts the quality of life and socio‐economic opportunities (Craig et al. 2009; Yaruss 2010). Neuroimaging findings have shown that the origin of this disorder can be traced to the central nervous system (Fox et al. 2000; Ingham et al. 2000; Watkins et al. 2008; Chang et al. 2009; Lu et al. 2010; Choo et al. 2011; Loucks et al. 2011; Chang and Zhu 2013; Chang et al. 2015). MEG (magnetoencephalography) and EEG (electroencephalography) studies have shown that overt speech‐related activities elicit aberrant brain activity (Salmelin et al. 2000; Beal et al. 2010, 2011). Despite such promising research, the temporal dynamics of exactly what transpires in neural processing immediately prior to or during the production of disfluent speech (any interruption in the forward flow of speech) remains poorly understood. Such information is critical to the understanding of stuttering, as disfluent episodes may manifest markedly distinct brain activation than fluent utterances (Jiang et al. 2012).

Different brain regions work in concert to produce speech (Hickok & Poeppel, 2007), so it is posited in this study that stuttering state could result from miscommunication within the speech motor network. Several scenarios could account for this miscommunication with one potential factor being sensorimotor “disintegration” (Guenther 2006; Beal et al. 2010; Sengupta et al. 2016b). It has been previously argued that temporal discoordination between articulatory and respiratory systems may lead to stuttering (Perkins et al. 1976). Similarly, motor timing deficits were observed in motor movements of adults who stutter (AWS) suggesting more generalized impairment of temporal coordination in their motor programming (Forster and Webster 2001; Olander et al. 2010; Etchell et al. 2014). Miscommunication in AWS could also arise due to cognitive processing load (Walla et al. 2004; Bosshardt 2006) or differences in phonological encoding (Byrd et al. 2012; Sasisekaran 2013; Pelczarski and Yaruss 2014, 2016) that possibly interact with the motor planning of speech.

Recent EEG and MEG studies have suggested that communication within functional brain networks in humans is accomplished by neural phase coherence, reflecting synchronous firing of neuronal population during patterned behavior in humans (Varela et al. 2001; Womelsdorf et al. 2007; Schroeder et al. 2008; Arnal et al. 2011; for reviews see Siegel et al. 2012; Fries 2015). Also, in animal studies phase coupling involving neuronal oscillations has been implicated in learning and memory (Lee et al. 2005; Tort et al. 2009). It is therefore expected that phase coherence subserves communication within brain networks during coordinated goal‐driven behaviors such as speech (Fries 2005) by organizing neural circuits (Schack et al. 2002). Indeed, distinct theta‐gamma coherence patterns have been shown to accompany motor adaptation and speech motor training in fluent adults (Perfetti et al. 2011; Sengupta and Nasir 2015, 2016a). The functional roles of neural oscillations in different stages of speech planning and production are not, however, well‐understood even in fluent speakers. Alpha and beta band activity are thought to be related to planning of overt speech, with alpha band more associated with attentional processing (Gehrig et al. 2012). These bands were found to play a role before word production in AWS (Jenson et al. 2014; Mock et al. 2016). An influential computational model (Giraud & Poeppel, 2012) suggested that theta and gamma oscillations are tied to the multi‐timescale, quasi‐rhythmic properties of speech. Also, gamma band is implicated in the effective processing of input and output generation (Schroeder and Lakatos 2009). Less research has been directed towards understanding how these oscillations are affected when speech is perturbed as in stuttering disfluencies. By comparing neural activity between disfluent and fluent utterances in AWS, one is potentially tapping into a stuttering state in distinction to a stuttering trait (Belyk et al. 2014). As the cortical state of AWS is often accompanied by higher neural overactivation (Budde et al. 2014), there could potentially be higher level of phase coherence during their typical speech.

Herein, the neural substrate of disfluency was examined using EEG and a behavioral paradigm that involves the production of phonologically challenging mainly nonword tokens designed to increase the likelihood of eliciting disfluency in a controlled environment. It is hypothesized that stuttering disfluencies will involve characteristic anomalies in neuronal oscillations and phase coherence patterns that precede speech onset reflecting neural miscommunication during motor planning within the speech motor network. As noted above, theta and gamma bands are involved in motor adaptation and motor memory and are expected to contribute to any anomaly(s) associated with stuttering. Involvement of other bands such as the alpha band cannot be ruled out if higher cognitive processes are implicated in eliciting disfluency.

## Methods

### Participants

Eight adults (2F (females); 26 ± 1.3 years; mean and SE) with persistent stuttering and eight fluent adults (3F; 22 ± 1.2 years) with no known history of speech or hearing disorders participated in this study. All participants were native English speakers, with no history of hearing concerns or speech/language disorders other than stuttering and received compensation for their participation in this study. Stuttering severity was assessed according to Systematic Disfluency Analysis (Gregory et al. 2003), a formal analysis tool used by speech‐language pathologists to quantify behavioral stuttering and speech disfluency patterns. This tool was specifically selected for this study as it accounts for multi‐component disfluencies, consideration of where in an utterance disfluency occurs, in the presence of physical tension within a specific disfluency, and more. Frequency of stuttering events (% syllables stuttered, or %SS) ranged from 8.5% to 24% (mean 15.4%). The Northwestern University Research Ethics Board approved all experimental procedures and written informed consent was obtained from all the participants.

### Stimuli

**Figure 1:**
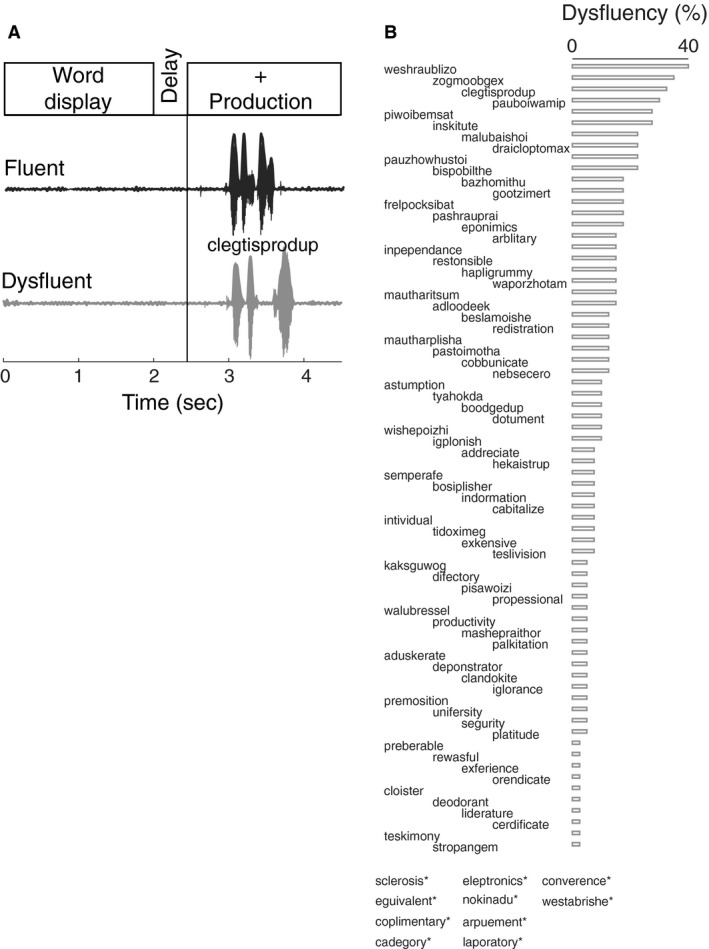
Behavioral paradigm for eliciting disfluency. (A) Speech motor task involved display of target utterances for 2 sec. After a 0.5 sec delay participants were prompted to read aloud the displayed utterances within a 2 sec long window. Speech waveforms corresponding to a fluent and disfluent version of an example target utterance, “clegtisprodup,” is shown below. (B). Disfluency score for all 80 target utterances used. Its range varied between 0 and 40%. 10 target utterances that did not elicit any disfluency are marked with asterisk.

A key challenge to studying cortical dynamics of stuttered speech lies in the elicitation of disfluency in controlled laboratory settings. AWS show a remarkable degree of variability in the production of speech disfluencies, from situation to situation and over time (see review in Constantino et al. 2016); often, they are fluent during repeated production of words (Salmelin et al. 2000; Sengupta et al. 2016b). Despite these challenges, there is evidence that phonological complexity can negatively impact motor stability in the fluent utterances of AWS, as well as performance on nonword repetition and other phonological processing tasks in children and AWS (Smith et al. 2010; Sasisekaran 2013; Pelczarski and Yaruss 2016). Nonwords that are longer, less word‐like, and contain later developing phonemes and consonant clusters are considered to be more difficult to produce, thus increasing the likelihood of stuttering. A list of 80 mainly nonword targets (Fig. 1B) were created, some of which were either real words (5 out of 80) or distorted slightly to form “word‐like” nonwords (e.g., teslivision) or “less word‐like” nonsense words (e.g., malubaishoi). Stimuli included 34 word‐like nonword tokens and 41 less word‐like nonwords that ranged in length from two to six syllables (Fig. 1B).All nonword stimuli were generated to contain combinations of longer phonological strings created with later‐developing phonemes and consonant clusters to increase phonological complexity in an effort to elicit stuttering.

### Experimental setup and task

All recordings were conducted in a soundproof booth. The speech task involved overt reading of the target tokens under continuous recording of EEG (Fig. 1A). The tokens were displayed for 2 sec, followed by a 0.5 sec delay, and a 2 sec long prompt for the participants to speak the word aloud. A real‐time Labview system (National Instruments) was used to display the speech tokens. Participants were instructed to speak immediately after the appearance of the prompt. There were a total of 80 speech tokens; each repeated five times, yielding a total of 400 stimuli read aloud by each participant. Each token was repeated not more than five times in order to reduce the fluency inducing effect due to adaptation. The stimuli were grouped in 40 blocks of 10 trials in each so that no stimulus was repeated twice in the same block. Participants were instructed to speak out the stimuli immediately upon prompting without contemplating their meaning or pronunciation.

### Disfluency score and acoustical analysis

Microphone outputs (Sennheiser ME‐66) were recorded using the Labview system at 40KHz. Each utterance was analyzed offline for the presence of disfluencies (specifically, part‐word repetitions, prolongations, or blocks). Trials in which the stimuli were uttered before the prompt signal and those whose utterance exceeded the 2 sec prompt window were discarded from the analyses (2.6% of all the trials). Thus, for each participant a disfluency score was obtained that was the percentage of nonfluent utterances over the total of 400 trials. The fluency scoring was done by a speech‐language pathologist that was later verified for reliability by two other experimenters.

The sound files were processed with customized Matlab routines. For each spoken utterance, the following three acoustical parameters were extracted in order to document the effect of disfluency on the acoustics of spoken utterances: speech‐onset time after the appearance of the production prompt, the duration of utterance, and the peak loudness (relative to the quiet phase). For each word, the average across participants over the disfluent and fluent trials was computed for each of these parameters.

### EEG acquisition

EEG data were obtained at a sampling rate of 512 Hz using a 64‐channel Brainvision system. The electrodes were mounted on an elastic cap using the standard 10–20 system of electrode placement, and electrical impedances of the scalp electrodes were kept below 10 kΩ. Only the scalp electrodes above the sensory and motor regions supporting the speech motor task were selected; therefore, electrodes over the occipital and extreme temporal regions were excluded. The remaining 38 electrodes indicated by gray circles in Figure 1E were analyzed. These sets of electrodes provided not only the lowest impedance, but were also less prone to muscle artifacts. Participants were instructed to minimize eye blinks and head movements during word production. Brief pauses of 1–2 sec between trials and 15–20 sec between blocks were inserted to avoid fatigue and muscle tension while minimizing head movements. The real‐time Labview system delivered a TTL (transistor‐transistor logic) pulse at the moment of the stimulus display and also at the production prompt in order to align EEG signals during offline analyses.

### Analysis of EEG powers and neural oscillations

#### Filtering

The EEG signals were extracted using Matlab‐based EEGLAB toolbox (Delorme and Makeig 2004) and band‐pass filtered offline between 0.75 and 55 Hz using a second‐order Butterworth filter. All trial ERP epochs were then time aligned at the first TTL pulse of the production prompt and re‐referenced at electrode Afz (Sengupta and Nasir 2015). A time window of 2500 msec preceding the appearance of the production prompt was used for the analysis reported in this article.

#### Artifact rejection

Stereotypical artifacts arising from eye movements, head movement, and muscular activity were removed by implementing the following steps. Epochs in which the scalp voltage at any of the electrode locations exceeded 75 *μ*V were excluded from further analysis. As a basis for further artifact rejection, the presence of aberrant temporal patterns and large negative kurtosis were detected. Muscle artifacts were eliminated by detecting spectral peaks that coincided with muscle activation and techniques based on independent component analysis (Olbrich et al. 2011). Overall, about 16% of the trials were excluded from further analyses due to artifact rejection.

#### Power and neural phase coherence

Each trial epoch was filtered using a fourth‐order Butterworth filter to obtain the instantaneous power over four EEG frequency bands. These bands were: theta (3–8 Hz), alpha (8–14 Hz), beta (14–30 Hz), and gamma (30–50 Hz). The Hilbert transformation was then used to obtain the instantaneous amplitude of the signal the square of which provided the power. Normalized power for each trial was obtained by dividing it by the overall power. Neural phase coherence between lower frequency bands (theta and alpha) and higher frequency bands (beta and gamma) was computed using the method described in Cohen (2008; see also Perfetti et al. 2011). The algorithm computes the degree of phase‐locking between the two bands that varies between 0 (perfect dysynchrony) and 1 (perfect synchrony). The algorithm requires the specification of a time window that was taken to be 800 msec long and slid by 10 msec in each step, as well as a 3 Hz frequency window slid by 1 Hz in each step.

#### Statistical bootstrapping

The subtraction method was used to obtain a difference signal between disfluent and fluent utterances. This paradigm has been widely used in imaging studies (Petersen et al. 1988; Power et al. 2014; McAvoy et al. 2016) that involve comparing brain states in two conditions that differed by a single feature (e.g., fluency vs. disfluency). Bootstrap sampling techniques (Efron 1982) corrected for family wise error (Pantazis et al. 2005) were used to derive statistical significances using *t*‐scores. For each electrode and for each participant a difference *t*‐score was obtained between fluent and disfluent utterances in the following way. For each word, the mean power (or phase‐coherence) for the disfluent and fluent utterances was first calculated. Their difference when averaged over all words gave the mean difference in power (or phase‐coherence) for each participant. These difference scores across participants were used to calculate the *t*‐score (mean over pooled standard deviation). It should be noted that for each participant only the tokens that elicited disfluency were included in this analysis. Recall that there were five trials per word, and the average number of disfluent trials per word was 1.46 ± 0.04.

Next, 4000 bootstrap samples of size 8 (from eight difference scores from AWS) were generated using sampling methods with replacement. A *t*‐score was calculated for each bootstrap sample. Thus, there were 4000 *t*‐score time series (or time‐frequency series) for each electrode. The maximum of the absolute *t*‐score overall electrodes and over the entire series was then used to obtain a distribution of maximum statistics (4000 such maximum from all bootstrap samples). The 99.5th percentile of this distribution (corresponding to *α *= 0.005) was taken as the critical *t*‐score. Regions (time‐frequency) for which the difference *t*‐score exceeded this critical value was considered to have shown a significant difference.

## Results

The goal of the study was to test whether anomalies in oscillatory brain dynamics precede disfluency in adults. The task consisted of brief display of the target token followed by a production prompt to cue overt reading (Fig. 1A). Figure 1B shows the distribution of disfluency scores for all the speech tokens. The utterances were categorized as stuttered disfluencies or fluent productions but were not sorted further into subcategories of disfluencies. There were 10 out of 80 tokens that did not elicit any disfluency across AWS, while 6 tokens had a disfluency score of at least 25%. The token “weshraublizo” elicited disfluency in 40% of the trials. The mean disfluency score over all nonwords across AWS was 10.0 ± 2.9% (mean and SE). Eight fluent adults tested in the same behavioral paradigm as controls had a mean disfluency score 1.3 ± 0.1%. AWS thus exhibited significantly more disfluency than the fluent participants (*t*_*14*_ = 2.93, *P *<* *0.02) and the complex stimulus set was effective in eliciting stuttering‐like episodes. It should be noted that the goal of this paper was to investigate the cortical state of disfluency in AWS. In a subsequent paper the cortical dynamics of stuttering trait will be investigated by comparing the fluent utterances of AWS with those from fluent adults.

**Figure 2:**
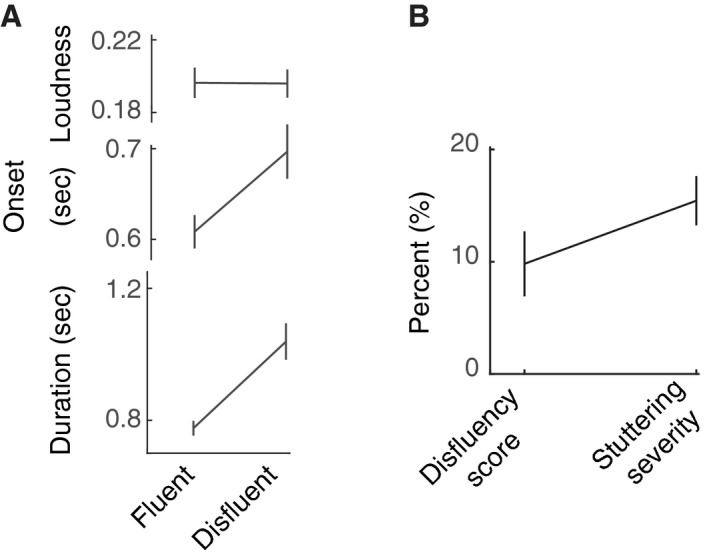
Disfluency and the acoustics of spoken utterances. (A) Significant effect of disfluency was observed for duration and production onset time after the appearance of the prompt, but not for peak loudness. (B) Disfluency score was comparable to stuttering severity.

Next in order to assess the effect of disfluency on the acoustics of the produced utterances, the average duration, loudness, and speech‐onset time were computed (Fig. 2A). The duration as well as the speech‐onset time after the appearance of the production prompt (see Methods) for the disfluent utterances was significantly longer (*t*_*136*_ = 4.41, *P *<* *2e‐05; *t*_*136*_ = 2.5, *P *<* *0.015) than the fluent utterances, while no significant differences in loudness levels were seen (*t*_*136*_ = 0.03, *P *>* 0*.95). It is worth noting that disfluency scores did not differ from stuttering severity (Fig. 2B; *t*_*14*_ = 1.5, *P > *0.15) suggesting that stuttering events were comparable in both experimental and conversational conditions.

**Figure 3:**
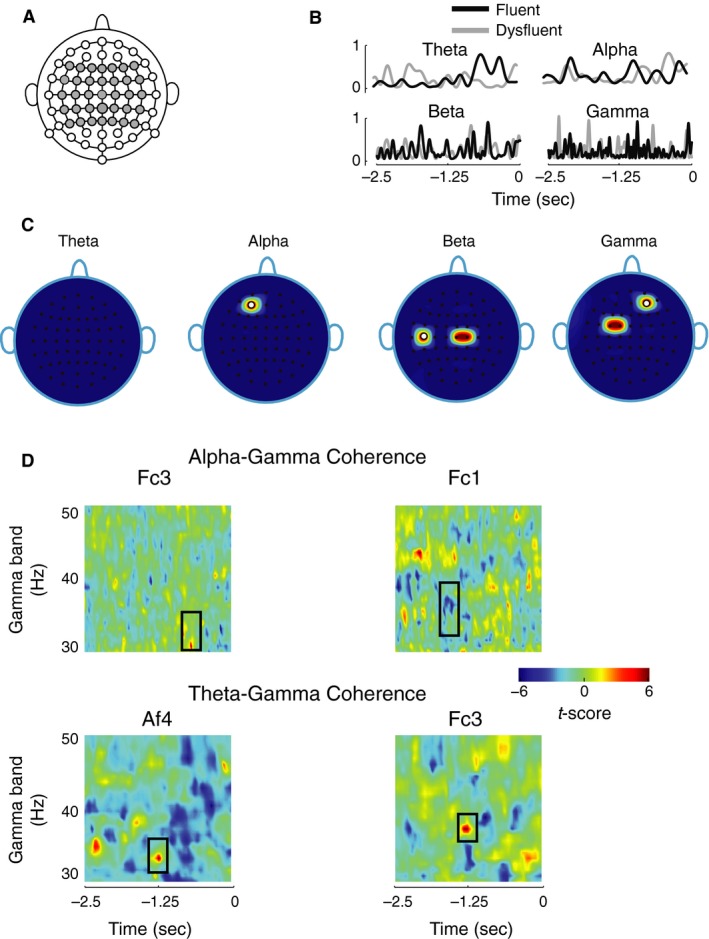
Neural activity shows anomaly preceding disfluency. (A) Scalp electrode locations used to record EEG from during the speech motor task are shown in gray. (B) Representative mean power traces for theta, alpha, beta, and gamma band are shown for fluent and disfluent utterances matched for words and participants. Only the portion of the power trace from the start of the display of a target utterance to the appearance of the production prompt is shown. Production prompt is at 0 sec. (C) Scalp electrode locations showing significant effect of disfluency on EEG power bands. Effects were observed for alpha band at electrode location Af3, for beta band at C2, C5, and Cz and for gamma band at Af4, Fc3, and Fc1. White circles denote electrode locations that showed significance rise in power preceding disfluency. At other electrode locations, power decreased significantly. (D) Phase coherence spectrograms based on *t*‐scores (color scale ranging from −6 to 6). Only phase coherence involving gamma band showed significant effect of disfluency. The ordinate of the spectrogram represents frequency range for the gamma band and the abscissa represent time, with 0 marking the appearance of the production prompt. The time axis includes the duration from the start of the display of a target utterance to the appearance of the production prompt. Alpha‐gamma phase coherence differences were observed at electrode locations Fc1 and Fc3, while theta‐gamma differences were found at Af4 and Fc3.

EEG brain signals were recorded from electrodes spanning the temporal, frontal, and parietal areas of the scalp during the entire epoch that started with the display of the target word and ended with its production (Fig. 3A). In order to examine the brain dynamics that precede disfluency, only the portion of the signal that started with the display of the token to the appearance of the production prompt was analyzed. This included the 2.5 sec long signal since the display of the token. The objective was to identify frequency bands and scalp electrode locations that showed significant differences between fluent and disfluent trials and, thus, isolate the neural processes related to disfluency itself. Figure 3B shows mean power from example disfluent and fluent trials matched for token‐type and participants for the theta, alpha, beta, and gamma bands.

Figure 3C plots electrode locations over the scalp that showed significant differences in *t*‐score (*P *<* *0.005, using bootstrapping and correcting for family wise error; see Methods) for EEG frequency bands associated with disfluency. Alpha band showed significant differences at left frontal electrode Af3, while beta and gamma bands had more significant electrodes mostly over the centro‐parietal scalp regions. Beta band showed significance differences at left central electrode C5, and more central electrodes Cz and C2. Gamma band differences were observed at left‐lateralized and anterior electrodes Fc3 and Fc1, and also at right frontal electrode Af4. The changes in power levels exhibited interesting patterns: At the two most frontal electrodes Af3 (alpha) and Af4 (gamma), there were significant rises in power levels. A significant rise was also observed for beta band at C5 (marked in white). For all other electrode locations, there were significant decreases in the power levels.

Next, the phase coherence at each of the identified electrodes that showed significant power differences was computed (Fig. 3D). The analysis revealed that only the gamma band phase coherence with alpha and theta bands was significant (*P *<* *0.005; see Methods). Alpha‐gamma coherence was significant around 1.35 and 0.6 sec prior to the production prompt, respectively, at electrodes Fc3 and Fc1. Electrode Af4 and Fc3 showed significant differences in theta‐gamma coherence around 1.25 sec and 1.2 sec prior to the production prompt, respectively. All phase coherence showed significant increase except alpha‐gamma coherence at Fc1, for which it was a decrease. Overall, all four electrodes involved in gamma band power exhibited significant differences in phase coherence either between alpha‐gamma or theta‐gamma band pairs.

## Discussion

The goal of this study was to identify whether anomalies in spectral power and spectral coherence precede disfluencies in AWS. Most studies of the neurology of stuttering have focused on brain activity during fluent speech or covert language processing due to the difficulty of studying neural function during moments of disfluency. Although several studies examined aberrant cortical activity preceding blocked or disfluent vocalization (Sowman et al. 2012; Vanhoutte et al. 2016), the neural anomalies that specifically give rise to stuttering remain elusive. As one‐step toward addressing this challenge, the current analysis focused on testing cortical dynamics in order to examine whether neural miscommunication within the speech motor network precedes stuttered speech. In a previous work (Sengupta et al. 2016b) a connection was shown between a lack of adaptation to an auditory perturbation and anomalous neural oscillations in AWS. As the anomalous neural oscillations preceded the onset of vocalizations, it provided support for the hypothesis examined here that disfluencies in AWS may be due to breakdowns in neural communication.

In terms of spectral power, it was found that distinct differences in alpha, beta, and gamma activity preceding disfluency at different electrode locations. It should be noted that previous studies found the engagement of the alpha and the beta band during the prespeech phase of AWS (Salmelin et al. 2000; Mersov et al. 2016). Similarly, there was alpha activity at a left‐frontal electrode location with beta/ gamma activity at parietal electrode locations and, additionally, gamma activity at right‐frontal electrode locations that were associated with subsequent instances of disfluency. The phase coherence analysis was also sensitive to these prespeech differences. Theta‐gamma coherence and alpha‐gamma coherence at the same electrode locations that showed gamma power differences were also altered prior to disfluency. In contrast, the beta band coherence with alpha and theta bands did not show changes before disfluencies relative to the fluent speech condition. Moreover, phase coherence primarily increased before disfluencies. This might be consistent with neural overactivation observed in stuttering state (Budde et al. 2014). It is normally assumed that the frequencies of the EEG power bands reflect spatial scales of the underlying brain networks subservient to them (von Stein and Sarnthein 2000; Bullmore and Sporns 2009; Hipp et al. 2011, 2012; Siegel et al. 2012). Thus, the anomalous neural activity observed here possibly involves brain network at multiple scales, ranging from the more local gamma and beta networks to larger and global networks, theta and alpha. Finding anomalies across these bands suggest that neural miscommunication precedes stuttered disfluencies and could be one of the pathological mechanisms underlying disfluencies.

A subtraction approach was used to assess disfluent state in AWS (Petersen et al. 1988; Power et al. 2014; McAvoy et al. 2016). In this procedure brain states are compared in two conditions differing by a single feature, such as comparing the brain states in fluent and disfluent conditions. By subtracting neural activity during the production of a fluent speech token from its disfluent version on a per participant basis, it is possible to isolate disfluency‐related brain activity while factoring out stimulus‐related complexity. Thus, fluent utterances in AWS served as their own control for assessing disfluency. The subtraction approach has its own limitations, namely that it ignores interactions at the neural level among various components of a cognitive task (Friston et al. 1996).

Does this aberrant brain activity reflect a preproduction stuttering state or something else, since the behavioral paradigm involved delayed response rather than conversational speech in which stuttering is typically elicited? The near absence of disfluency in fluent participants points to the fact that the stimulus set effectively elicited disfluency in AWS. Also, the average stuttering severity was comparable to the average disfluency score. In a similar behavioral paradigm differences in neural activity prior to overt speech production was also observed (Salmelin et al. 2000) even for fluent utterances. The anomalous phase coherence observed in the present study preceded speech onset at least by 0.5 sec raising the possibility that the anomaly in question is related to cognitive processes involved in speech, not exclusively motor preparation per se. Stuttering may involve a core sensorimotor deficit interacting with various cognitive processes such as phonological encoding and memory. Evidence suggests that both the phonological encoding and the phonological memory of children and adults who stutter are less robust than typically fluent peers (Byrd et al. 2012; Sasisekaran 2013; Pelczarski and Yaruss 2014, 2016). Furthermore, stuttering may arise due to deficits in word recognition (Wells and Moore 1990; Hubbard and Prins 1994). In theories of spoken word retrieval (Levelt 2001) access to semantic information is believed to interact with phonological encoding, consequently, an interaction between the motor system and these processes could thus serve as an information bottleneck eliciting disfluency (Smith et al. 2010). The involvement of theta‐gamma phase coherence could indeed point to such memory mediated processes involving motor memory contributing to disfluency (Fell and Axmacher 2011; Perfetti et al. 2011). Likewise, the observed alpha band phase anomaly suggests an attentional component might contribute to elicitation of disfluency (Foxe and Snyder 2011). The timing of the anomalies further implies a cascading process that could start with attentional miscommunication interacting with phonological planning and memory access. It is of great interest to find out whether the brain networks giving rise to these phase anomalies include sensorimotor cortical areas to determine the extent to which core sensorimotor deficits overlaps with such cognitive processes. This will add to the growing literature on neural activation differences in various aspects of language processing in adults and children who stutter (Weber‐Fox 2001; Maxfield et al. 2010, 2012).

This is the first study to directly probe neural phase coherence that might be associated with disfluent utterances, but there are several caveats. First, even though the stimulus set was able to evoke disfluency in almost all participants, the occurrence of disfluencies was relatively small (~10%), making it imperative to use many different tokens. Nonetheless, the stringent bootstrapping approach confers confidence that distinct neural activity preceded disfluent utterances. Second, the mean disfluency score across AWS did not differ from the mean stuttering severity, suggesting that even in isolated experimental condition the behavioral paradigm elicited similar level of disfluency observed in conversational setting. Nevertheless, there is a greater need to test out the behavioral paradigm using a larger sample for a more robust validation of the behavioral paradigm and the findings reported here. Third, the list of tokens and the experimental setting could lack ecological validity; participants did not speak in full sentences and speaking in isolation in the laboratory setting was not a natural speaking environment. It is well known that AWS show markedly different speech behavior in natural settings. Therefore, to study the neural correlates of speech disfluency, it would be desirable to study disfluencies with more naturalistic utterances.

Moving forward, the next step is extending these analyses to the source level to locate the brain areas involved. Now that it has been demonstrated which power levels and phase coherence are involved in disfluency, it is desirable and possible in future studies to investigate their underlying neural sources and the pattern of their interaction at the source level. Also, carrying out studies of neural oscillation in speech development will provide key insights into the nature of speech disfluency, motivating novel diagnostics and therapeutic techniques in dealing with the disorder.

## Conflict of Interest

None declared.
